# Phage therapy for multidrug-resistant pulmonary infections: bridging precision matching, lung delivery, host immunity, and clinical translation

**DOI:** 10.3389/fcimb.2026.1968526

**Published:** 2026-09-22

**Authors:** Ran Xiao, Yang Hao, Yadong Gong, Anjun She, Qingqing Ma, Mu Lin, Yubing Guo, Ting Jiang

**Affiliations:** 1 Central Laboratory, Department of Clinical Laboratory, Guizhou Aerospace Hospital, Zunyi, China; 2 Department of Internal Medicine, University Medical Center Hamburg-Eppendorf, Hamburg, Germany; 3 Hamburg Center for Kidney Health (HCKH), University Medical Center Hamburg-Eppendorf, Hamburg, Germany

**Keywords:** airway microbiome, clinical translation, drug-resistant bacteria, immune regulation, microbiome modulation, phage therapy, pulmonary delivery

## Abstract

Chronic pulmonary infections caused by multidrug-resistant (MDR) bacteria are a growing global health problem, and the declining effectiveness of conventional antibiotics increases the need for alternative antimicrobial strategies. Unlike broad-spectrum antibiotics, bacteriophages are highly specific, and their use therefore offers not only antibacterial activity but also the possibility of reshaping the airway microbial ecosystem in a targeted manner. In this review, we reframe phage therapy as a precision microbiome-modulating strategy for chronic pulmonary infections. We first review precision pathogen identification and phage-host matching, then discuss formulation engineering for pulmonary delivery, and then examine the interactions between phages and host immune barriers. We further analyze how phage therapy may selectively deplete MDR pathogens while sparing the commensal microbiota and how it may remodel the airway microbial ecosystem, and we critically evaluate the clinical evidence, distinguishing preclinical data, case-based evidence, observational studies, and controlled trials. By integrating these dimensions, we identify the main scientific and technical challenges and evidence gaps that currently limit clinical implementation, and we discuss strategies that could support the development of safe, effective, and clinically applicable phage-based therapies for chronic pulmonary infections.

## 1 Introduction

Pulmonary infections remain a leading cause of global morbidity and mortality, and the spread of multidrug-resistant (MDR) organisms has severely compromised conventional antibiotic therapy. The World Health Organization has classified antimicrobial resistance as a global threat (Reynolds et al., 2022). MDR and extensively drug-resistant (XDR) Gram-negative strains continue to increase in hospitals, environmental sources, and communities; pneumonia is the most common presentation and carries high mortality. In Vietnam, lower respiratory tract isolates were predominantly *Acinetobacter baumannii* (49.6%), *Pseudomonas aeruginosa* (21.0%), and *Klebsiella pneumoniae* (18.6%), and MDR and carbapenem resistance in *A. baumannii* reached 96% and 95%, respectively (Dung et al., 2025). In a Saudi Arabian ICU, *K. pneumoniae* (34.0%), *Pseudomonas* spp. (27.0%), and *Acinetobacter* spp. (12.0%) predominated, with high β-lactam and carbapenem resistance (Almouwlid et al., 2025). In chronic pulmonary diseases such as cystic fibrosis (CF) and chronic obstructive pulmonary disease (COPD), persistent biofilm-associated infections are particularly intractable. *P. aeruginosa* drives pulmonary function decline in CF; MDR, XDR, and pandrug-resistant (PDR) phenotypes accounted for 19%, 16%, and 24% of 85 strains, respectively (Ali and McDermott, 2025). Among acute COPD exacerbations, Gram-negative bacteria predominated (93.1%), with *A. baumannii* complex (23.7%), *P. aeruginosa* (21.4%), and *K. pneumoniae* (12.6%) most common (Wang et al., 2026). In cirrhosis patients, lower respiratory tract infections comprised 6.4% of cases, with MDR and XDR accounting for 31.4% and 22.6%, respectively (Varghese et al., 2025). Biofilms enhance bacterial tolerance and immune evasion, drive recurrent infections, and resist clearance by current drugs, reinforcing the need for new antimicrobial strategies.

Bacteriophages, obligate bacterial viruses, have several properties suited to treating drug-resistant pulmonary infections: high target specificity, self-replication, and precise lysis of drug-resistant bacteria. These properties are context dependent. Phages can spare the normal microbiota, amplify in parallel with bacterial proliferation, and penetrate and kill bacteria within biofilms, but the extent of each depends on host susceptibility, bacterial density, local physicochemical conditions, immune clearance, and the characteristics of the individual phage (Vaezi et al., 2024).In addition, phages show minimal toxicity to eukaryotic cells. *P. aeruginosa* phage PEV has been formulated for nebulization; combined with ciprofloxacin as an inhalable powder, it retained stability and showed bactericidal activity in a mouse pulmonary infection model (Chan and Chang, 2022). Antibiotic synergy with phages, immune-phage interactions in animal models, and pharmacokinetic and pharmacodynamic data together support their therapeutic use (Vaezi et al., 2024).

Translating phage therapy from the laboratory to the clinic faces several bottlenecks: complex interactions with the host immune system, formulation engineering challenges in pulmonary delivery, and a lack of standardized clinical protocols. Antibodies produced by the immune system can neutralize phage activity and compromise efficacy. Maintaining phage viability during nebulization or dry powder inhalation and achieving effective pulmonary deposition remain difficult for delivery systems. As an analogous, though not phage-specific, delivery approach, one study encapsulated nanoparticles within liposomes to improve antibiotic selectivity (Fakhoury et al., 2025). The absence of standardized protocols for phage selection, dosing, route of administration, and efficacy assessment has also impeded clinical translation.

Basic research and clinical cases have now shown both the potential and the limits of phage therapy in pulmonary infections, and a systematic synthesis of these findings is needed. Published reviews have covered the major aspects of this field: Iszatt et al. (2021) surveyed the evidence against MDR pathogens, including pulmonary delivery, animal efficacy, and human trials; the review by Vaezi et al. (2024) in the European Respiratory Review provided a comprehensive overview of phage therapy for lower respiratory tract infections, covering phage-antibiotic synergy, immune-phage interactions, pharmacokinetics/pharmacodynamics, and clinical translation; the systematic review by Sarkodie-Addo et al. (2025) and the more recent reviews by Terzi et al. (2026) and Sukri et al. (2026) have updated the clinical evidence landscape. Integrating these advances and defining the current status, advantages, bottlenecks, and future directions of phage therapy in MDR bacterial pulmonary infections should help guide basic research and clinical translation.

This review differs from the works above in three respects. First, the conceptual framework: we reframe phage therapy as a precision microbiome-modulating strategy—the central question is not whether phages can kill drug-resistant bacteria, but how phages can selectively deplete MDR pathogens and steer the airway microbial ecosystem back toward a health-associated state—and we use this framework to integrate the four dimensions of matching, delivery, immunity, and clinical translation, rather than surveying them topic by topic. Second, Section 5 analyzes the interaction between phage therapy and the respiratory microbiome (selective pathogen depletion with commensal sparing, ecological niche remodeling and community succession, and the host-microbiome immune axis) as a dedicated theme, which prior respiratory reviews have not treated as a standalone section. Third, we stratify the clinical evidence strictly (preclinical, case-based, observational, and controlled trials), verify the primary reports of each cited human case, and incorporate the primary case-series evidence published in 2025–2026.

The scope of this review is focused on pulmonary applications. Throughout, we distinguish evidence obtained directly from pulmonary or respiratory models (e.g., nebulized or inhaled delivery, lung infection models, and pulmonary clinical cases) from evidence extrapolated from systemic or non-pulmonary infection models, and we state the source model whenever findings are generalized to the lung. Statements about phage properties and clinical efficacy are likewise qualified by the experimental or clinical context and by the level of the supporting evidence.

A systematic literature search was performed in PubMed, Web of Science, and Google Scholar for English-language articles published from database inception through August 6, 2026 (the date of the final search), using combinations of the following keywords: phage therapy, bacteriophage, multidrug-resistant, extensively drug-resistant, pulmonary infection, lung infection, inhalation, nebulization, dry powder inhaler, biofilm, host immunity, airway microbiome, and case report. Reference lists of retrieved articles and relevant reviews were screened for additional studies. Studies were included if they reported primary preclinical or clinical data on phage therapy for bacterial pulmonary infections, or directly relevant methodological advances in matching, formulation, or immunology; secondary sources were cited only when a primary report was unavailable.

## 2 Precision pathogen typing and phage matching strategies

### 2.1 Molecular basis of phage-host interaction and matching principles

Phage infection of bacteria begins with adsorption, which depends on high-affinity binding between receptor-binding proteins (RBPs) on phage tail fibers or baseplates and specific receptors on the bacterial surface. These receptors include the O-antigen or core polysaccharide of lipopolysaccharide (LPS), repeating units of capsular polysaccharide (CPS), outer membrane proteins (e.g., OmpC, OprM), flagellin, or pili. RBP-receptor binding typically involves electrostatic interactions, hydrogen bonds, and hydrophobic forces, and specificity is determined by the conformation of the RBP and the spatial structure of the receptor epitope. For example, LPS is a key target recognized by multiple *P. aeruginosa* phages, whereas the K2 capsular polysaccharide is the primary receptor for *K. pneumoniae* phage Kpp-9 (Wang et al., 2025). After adsorption, conformational changes in the baseplate or tail fibers trigger genome injection; this step requires enzymatic degradation of the bacterial cell wall and membrane penetration. Different phages use distinct injection strategies: Podoviridae penetrate the cell wall directly through tail tubes, whereas Siphoviridae rely on tail fiber contraction to drive DNA injection. Receptor density and distribution on the bacterial surface directly influence adsorption efficiency. When bacteria acquire mutations that cause LPS glycosylation changes or O-antigen loss, for instance, the RBP recognition site is masked and adsorption efficiency falls by several orders of magnitude.

The diversity of surface receptors among bacterial strains makes phage host ranges narrow, so precision typing is a prerequisite for matching. Traditional serotyping (e.g., K-antigen typing for *K. pneumoniae*) and molecular typing (e.g., multilocus sequence typing (MLST) and whole-genome sequencing (WGS)) provide preliminary strain lineage information but predict phage infectivity poorly. Targeted sequencing of receptor gene clusters (e.g., LPS synthesis genes, CPS gene clusters) and high-throughput screening of phage infection phenotypes have now been incorporated into matching algorithms. For *A. baumannii*, for example, the broad-host-range phage Abp95 lysed multiple sequence types of carbapenem-resistant strains, suggesting that receptor genotype-based matching can extend the applicability of phages (Huang et al., 2023). For *P. aeruginosa*, phages PaCCP1 and PaCCP2 isolated from wastewater lacked virulence or antibiotic resistance genes by genomic analysis and showed lytic activity against multiple MDR strains, further supporting molecular feature-based matching in therapeutic phage selection (Parra et al., 2024). Machine learning models that correlate receptor genotypes with phage lysis spectra can guide the selection of highly lytic phages from clinical specimens or environmental libraries, a step toward precision therapy. Phage receptor-binding proteins recognize specific bacterial surface receptors, including lipopolysaccharide, capsular polysaccharide, and outer membrane proteins, thereby initiating adsorption, genome injection, and bacterial lysis (**Figure 1**).

**Figure 1:**
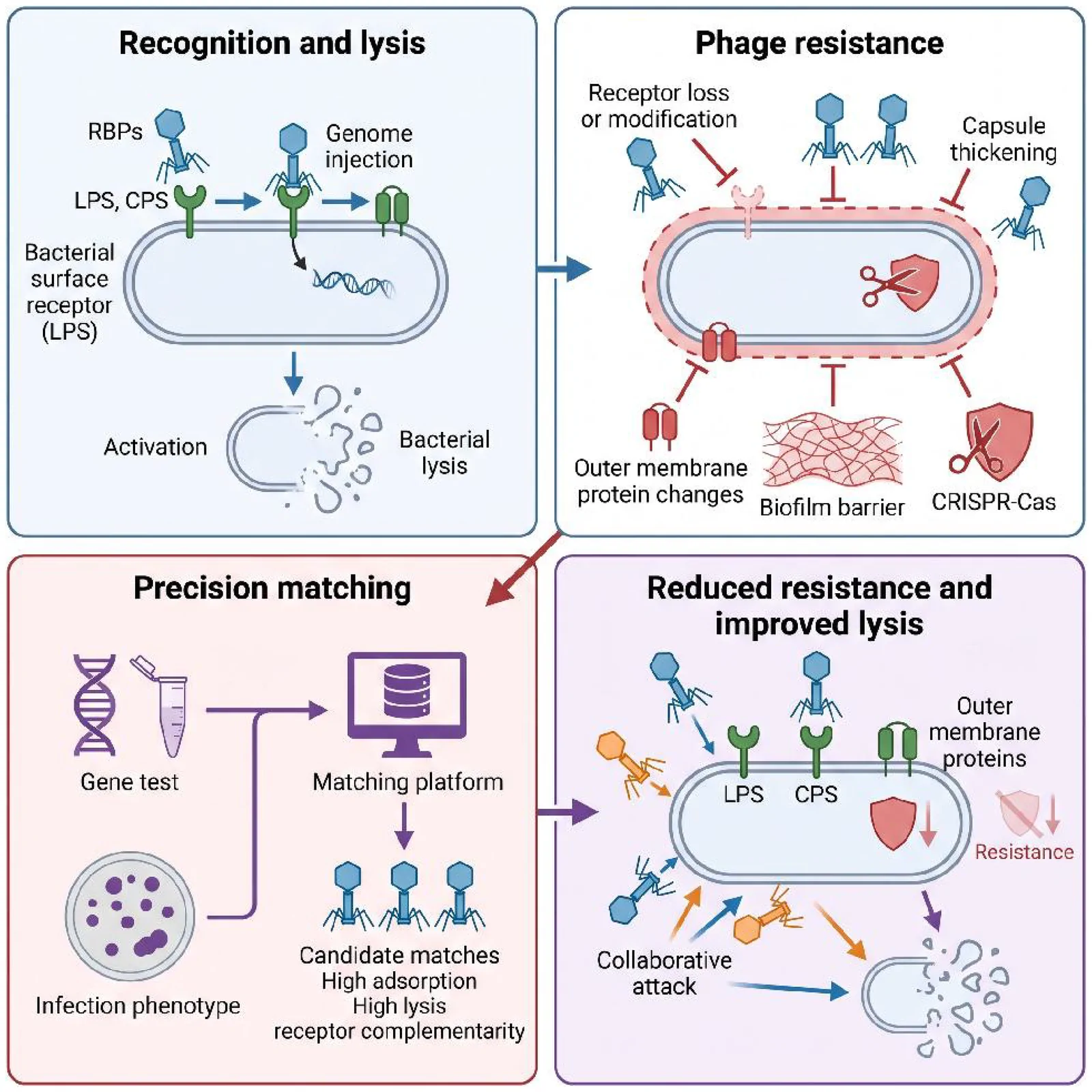
Molecular mechanisms underlying phage–host recognition, precision matching, and cocktail-mediated resistance suppression. Phage receptor-binding proteins recognize specific bacterial surface receptors, including lipopolysaccharide, capsular polysaccharide, and outer membrane proteins, thereby initiating adsorption, genome injection, and bacterial lysis. Bacteria can evade infection through receptor loss or modification, capsule thickening, outer membrane remodeling, biofilm formation, and CRISPR-Cas-mediated degradation of phage DNA, resulting in impaired adsorption and infection. Precision matching integrates bacterial receptor genotypes, genomic information, infection phenotypes, and phage characteristics to select phages with high adsorption efficiency, strong lytic activity, and complementary receptor specificity. Phage cocktails targeting distinct bacterial receptors impose multi-target pressure, increase the evolutionary barrier to resistance, reduce the emergence of resistant bacteria, and enhance bacterial clearance. Dynamic monitoring of receptor variation and phage susceptibility enables timely optimization of cocktail composition, thereby improving the durability and therapeutic efficacy of phage therapy against multidrug-resistant pulmonary pathogens.

### 2.2 Impact of bacterial resistance mechanisms on phage matching

Bacterial resistance mechanisms determine how well phage receptor-binding proteins are recognized, so the matching process must assess receptor accessibility and structural variation on the surface of drug-resistant strains. These mechanisms differ substantially among the major pulmonary pathogens. In *P. aeruginosa*, resistance commonly arises from structural modification of LPS, such as O-antigen loss or altered glycosylation of the core polysaccharide, which masks phage RBP adsorption sites. Mutations in the outer membrane protein OprM or overexpression of efflux pumps (e.g., MexAB-OprM) can also reduce effective phage binding (Sawant et al., 2026). *K. pneumoniae* mainly evades phage recognition through capsular polysaccharide (CPS) serotype switching or mutations in the K-antigen gene cluster; capsular thickening in K2-type strains can physically impede phage penetration, and mutations in the capsule depolymerase target of K57-type strains abolish phage lysis (Wang et al., 2025). *A. baumannii* resistance is more complex, involving loss or point mutations of outer membrane proteins (e.g., CarO) and phosphoethanolamine modification of lipooligosaccharide (LOS). These changes can impair phage adsorption and alter cell surface charge, which may disrupt electrostatic interactions with phage tail fiber proteins (Dehbanipour and Ghalavand, 2022). In mycobacteria (e.g., *Mycobacterium abscessus*), phage resistance tracks with structural remodeling of the cell wall mycolic acid layer; for example, upregulation of MmpL transporters alters cell wall permeability, while the CRISPR-Cas adaptive immune system can target and degrade invading phage DNA; together these constitute a dual defense barrier (Ferreiro-Posse et al., 2026).

Building on these mechanisms, researchers are developing predictive matching algorithms that combine resistance genotypes with phage infection phenotypes to select highly lytic phages from clinical specimens or environmental sources. Against carbapenem-resistant *A. baumannii*, for example, phages ΦZC2 and ΦZC3 isolated from hospital wastewater lysed different clinical isolates, and ΦZC3 protected lung cells from infection in an A549 cell model, suggesting it is a candidate for treating pulmonary infections (Essam et al., 2025). Against carbapenem-resistant *K. pneumoniae*, the broad-host-range phage phiA85 lysed strains of 13 different KL types and 12 sequence types, and combining it with imipenem or ciprofloxacin reduced mortality in a mouse pneumonia model (Li et al., 2025). These results indicate that the matching process must account for the molecular characteristics of drug-resistant strains. Phage combination regimens tailored to specific resistance profiles can prevent treatment failures caused by receptor variation and improve the clinical prospects of phage therapy for MDR bacterial pulmonary infections.

### 2.3 Phage cocktail strategies and resistance evasion

In single-phage therapy, bacteria can rapidly develop resistance through point mutations, horizontal gene transfer, or epigenetic modifications. *P. aeruginosa*, for example, can escape lysis entirely through LPS O-antigen loss or glycosylation changes that affect phage RBP targets, with adsorption efficiency falling sharply (Wang and Kirienko, 2026). Such resistance mutations erode the durability of single-phage therapy and push clinical practice toward multi-target combinations. Phage cocktails limit bacterial escape routes by combining phages that recognize different receptors (e.g., LPS, outer membrane proteins, capsular polysaccharide) or use different lytic mechanisms (e.g., holin-endolysin systems, polysaccharide depolymerases). In *K. pneumoniae*, cocktails that target both K-antigen and O-antigen lower the frequency of resistance mutations, because altering both receptors simultaneously is costly and often produces capsular synthesis defects or compromised cell wall integrity (Gou et al., 2025). In a mouse pulmonary infection model, a cocktail of phages GZ7 and GZ9 inhibited bacterial growth, reduced the emergence of resistant bacteria, and lowered pulmonary bacterial burden and inflammatory cytokine levels; efficacy matched single-phage therapy with a lower risk of resistance (Gou et al., 2025).

Resistance mutations that arise under cocktail pressure usually carry fitness costs. When *P. aeruginosa* loses LPS O-antigen under phage pressure, for example, outer membrane permeability increases and susceptibility to polymyxins returns. When *K. pneumoniae* evades capsule-targeting phages, K-antigen gene cluster mutations thin the capsule, reducing secretion of virulence factors (e.g., siderophores) and weakening resistance to neutrophil phagocytosis (Wang et al., 2025). This “resistance-virulence trade-off” gives phages and antibiotics a molecular basis for synergy: cocktails can drive bacteria into a hypovirulent state that conventional antibiotics can then eliminate. In a mouse acute pulmonary infection model, phage vB_Kpn_FOPMU1 combined with cefotaxime achieved 100% survival versus 60% for single-phage therapy, and the combination improved pulmonary histopathology (El-Din et al., 2025). Resistance mutations can also alter population behavior; some *A. baumannii* strains under phage pressure upregulate biofilm formation genes to block phage penetration, yet this simultaneously strengthens the biofilm barrier against antibiotics, creating a therapeutic dilemma (Dehbanipour and Ghalavand, 2022). Cocktail strategies should therefore integrate resistance surveillance data and adjust phage combinations in real time (e.g., replacing ineffective components or introducing engineered phages) to counter emerging resistance and preserve long-term efficacy (Boroujeni et al., 2024).

## 3 Formulation engineering optimization of pulmonary delivery systems

### 3.1 Challenges and advances in nebulization inhalation delivery technology

Pulmonary drug delivery is the preferred route for treating lower respiratory tract infections because it brings therapeutic agents directly to the site of pathology, raises local drug concentrations, and limits systemic side effects. Conventional nebulizers (e.g., jet or ultrasonic), however, generate shear forces, thermal effects, and interfacial stress that can damage phage particles and reduce their biological activity; this is the main obstacle in pulmonary phage delivery (Encinas-Basurto et al., 2025). Mechanical stress during nebulization is the primary cause of phage titer loss, and phage morphotypes differ in tolerance, with Myoviridae and Siphoviridae showing different stability during nebulization (Encinas-Basurto et al., 2025). A high-frequency acoustic nebulization platform (HYDRA) minimizes denaturation of phages and lysins through precise control of acoustic frequency and energy, achieving less than 0.1 log_10_ titer loss and producing aerosol particles in the ideal 1-5 μm inhalation range for deep-lung deposition (Encinas-Basurto et al., 2025). Formulation also helps protect phage integrity during nebulization. Stabilizers such as sugars (lactose, trehalose) and amino acids, together with the dispersibility-enhancing excipient leucine, preserve phage structure (Pathak et al., 2025). In spray-dried powder formulations of *P. aeruginosa* phages, hydrolyzed gelatin gave the best protection, with only a 0.6 log_10_ titer decrease during spray drying; adding 5% leucine or trileucine as dispersion enhancers produced an emitted dose (ED) of 90% and a fine particle fraction (FPF) of 80-86% (Pathak et al., 2025). Optimizing nebulization technology and formulation composition can thus limit the activity loss seen in pulmonary phage delivery, an essential step toward clinical use. Pulmonary drug delivery is the preferred route for treating lower respiratory tract infections because it brings therapeutic agents directly to the site of pathology, raises local drug concentrations, and limits systemic side effects (**Figure 2**).

**Figure 2:**
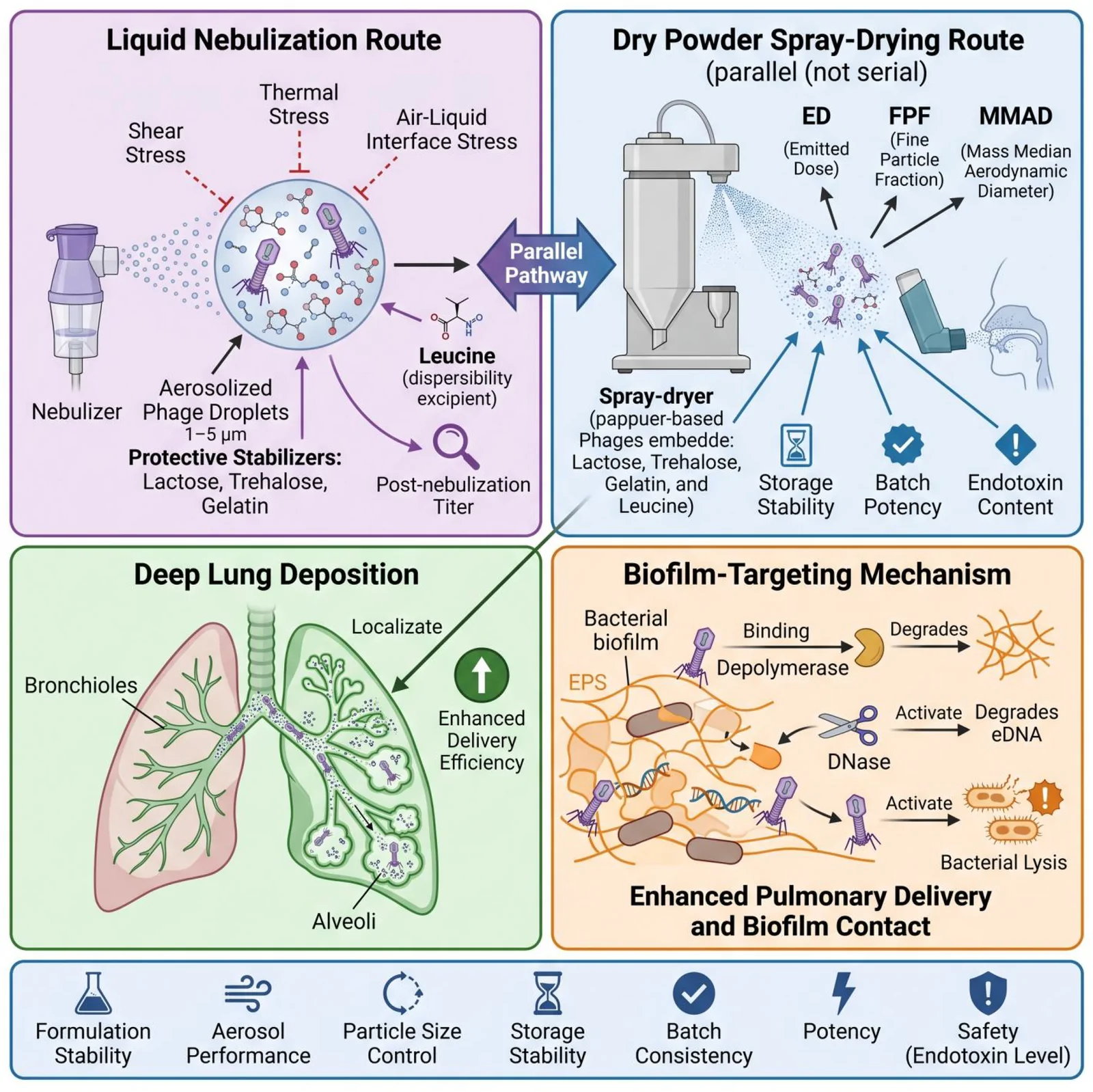
Formulation engineering strategies for pulmonary phage delivery. Four panels summarize the strategies covered in this chapter. (Top-left, purple) Liquid nebulization generates 1–5 μm aerosol droplets, protected by lactose, trehalose, and gelatin, with leucine as a dispersibility enhancer; shear, thermal, and air–liquid interfacial stresses (red dashed lines) are the principal activity-loss factors, monitored by post-nebulization viable titer. (Top-right, blue) Spray-drying—an alternative, non-sequential route—embeds phages with the same excipients into dry powders, evaluated by emitted dose (ED), fine particle fraction (FPF), and mass median aerodynamic diameter (MMAD). (Bottom-left, green) Deep deposition in bronchioles and alveoli. (Bottom-right, orange) Biofilm targeting: phage polysaccharide depolymerases degrade the EPS matrix, DNase degrades extracellular DNA, and phages lyse entrapped bacteria. The bottom strip lists seven translational quality attributes—viable titer, ED, FPF, MMAD, storage stability, endotoxin, and batch potency—linking the two routes to biofilm penetration.

### 3.2 Long-term stability of inhalable powder formulations

Liquid nebulization and spray-dried dry powder delivery are distinct formulation routes with different stability and aerosolization challenges; the preceding section addressed liquid formulations for nebulization, whereas this section focuses on the long-term stability of dry powder formulations.

Developing phage dry powder inhalers that stay stable at room temperature is a key step toward clinical translation, because liquid formulations lose activity during storage and powders do not. In a four-year longitudinal study, spray-dried phage powders with lactose as stabilizer and leucine as dispersant lost approximately 0.97 log_10_ titer of activity when stored at 4 °C and 15% relative humidity, indicating good storage stability (Encinas-Basurto et al., 2025). The same study raised a separate concern: the fine particle fraction of the powder fell from an initial 50-60% to 27-44% after prolonged storage, showing that the physicochemical properties of dry powder formulations change over time and may produce inconsistent clinical efficacy (Encinas-Basurto et al., 2025). Formulations with a high lactose proportion (70-80%) preserved phage activity better, suggesting that the stabilizer-to-excipient ratio is a key determinant of long-term stability (Encinas-Basurto et al., 2025). Excipients also differ in their effects on phage stability. In a comparison of trehalose, lactose, mannitol, gelatin, and hydrolyzed gelatin, the two gelatins showed virtually no decline in phage titer after 12 weeks at 4 °C, the best long-term stability (Pathak et al., 2025). Inhalable phage powder development must therefore weigh how stabilizer type, proportion, and storage conditions affect both biological activity and aerosol performance. Future work should examine new stabilizer combinations and drying processes that can deliver powders combining high stability with good inhalability for clinical use.

Key translational quality attributes that should be reported for inhalable phage products include post-nebulization viable titer, emitted dose, fine particle fraction, mass median aerodynamic diameter, storage stability, endotoxin content, and batch-to-batch potency.

### 3.3 Targeted delivery and biofilm overcoming strategies

Chronic pulmonary infections are often associated with biofilms formed by pathogens such as *P. aeruginosa* and *K. pneumoniae*. The biofilm matrix acts as a physical barrier that impedes phage penetration, and by lowering local pH and restricting nutrients it also weakens phage lytic activity, diminishing therapeutic efficacy (Das and Kaledhonkar, 2024; Rahimi et al., 2023). Formulation engineering must therefore improve aerosol performance and phage diffusion within biofilms at the same time. *K. pneumoniae* phage MKP-1 degrades pre-formed biofilm structures, an activity that tracks with the polysaccharide depolymerase and other lytic enzymes encoded in its genome (Das and Kaledhonkar, 2024). Likewise, the genome of phage SAKp02 carries holin, endolysin, and depolymerase genes whose products drive bacterial lysis and biofilm disruption; after 4 hours of treatment, biofilm biomass fell more than threefold and 99% of viable bacterial cells were eliminated (Chakraborty et al., 2024). To breach the biofilm barrier, researchers have designed nanocarriers and composite formulations containing matrix-penetrating enzymes (e.g., polysaccharide depolymerases, deoxyribonucleases) that increase local phage bioavailability (Wacnik et al., 2026). Polysaccharide depolymerases expressed by engineered phages, for instance, degrade the K57 capsule of *K. pneumoniae* and lower bacterial burden in mouse lungs, an effect that combines targeted delivery with biofilm disruption (Chakraborty et al., 2024). Nanocarrier technologies such as solid lipid nanoparticles and polymeric nanoparticles can also protect phage activity during delivery and, after surface modification, direct phages to biofilm regions (Encinas-Basurto et al., 2025). Together, these strategies can improve the efficacy of phage therapy in chronic pulmonary infections and offer a route to treating biofilm-associated drug-resistant infections.

## 4 Regulatory mechanisms of host immune barriers

### 4.1 Interaction between phages and the innate immune system

After nebulization and entry into the lungs, phages are recognized and cleared by innate immune cells such as alveolar macrophages and neutrophils; this is the primary immune barrier that determines phage efficacy. Pattern recognition receptors on alveolar macrophages (e.g., Toll-like receptors) sense phage nucleic acid or protein components. Unmethylated CpG DNA motifs of phages, for instance, are recognized by TLR9, which activates intracellular NF-κB and IRF signaling, induces type I interferons and other inflammatory cytokines from macrophages, and in turn alters the local immune microenvironment and bacterial clearance efficiency (Rodriguez-Gonzalez et al., 2024). Phospholipids and proteins in pulmonary surfactant (e.g., SP-A, SP-D) can also bind phage capsid proteins, promoting macrophage phagocytosis through opsonization or destabilizing the phage particle directly. This interaction shortens the effective residence time of phages in the alveolar space and may accelerate their clearance through complement activation (e.g., C3b deposition), creating a dynamic interplay between innate immunity and phage efficacy (Flint et al., 2023). Defining how innate immune cells recognize phages (e.g., intracellular signaling networks) is therefore a necessary prerequisite for designing targeted dosing strategies (e.g., pulsed dosing or immunosuppressive pretreatment) that overcome these barriers (Terzi et al., 2026). After nebulization and entry into the lungs, phages are recognized and cleared by innate immune cells such as alveolar macrophages and neutrophils; this is the primary immune barrier that determines phage efficacy (**Figure 3**).

**Figure 3:**
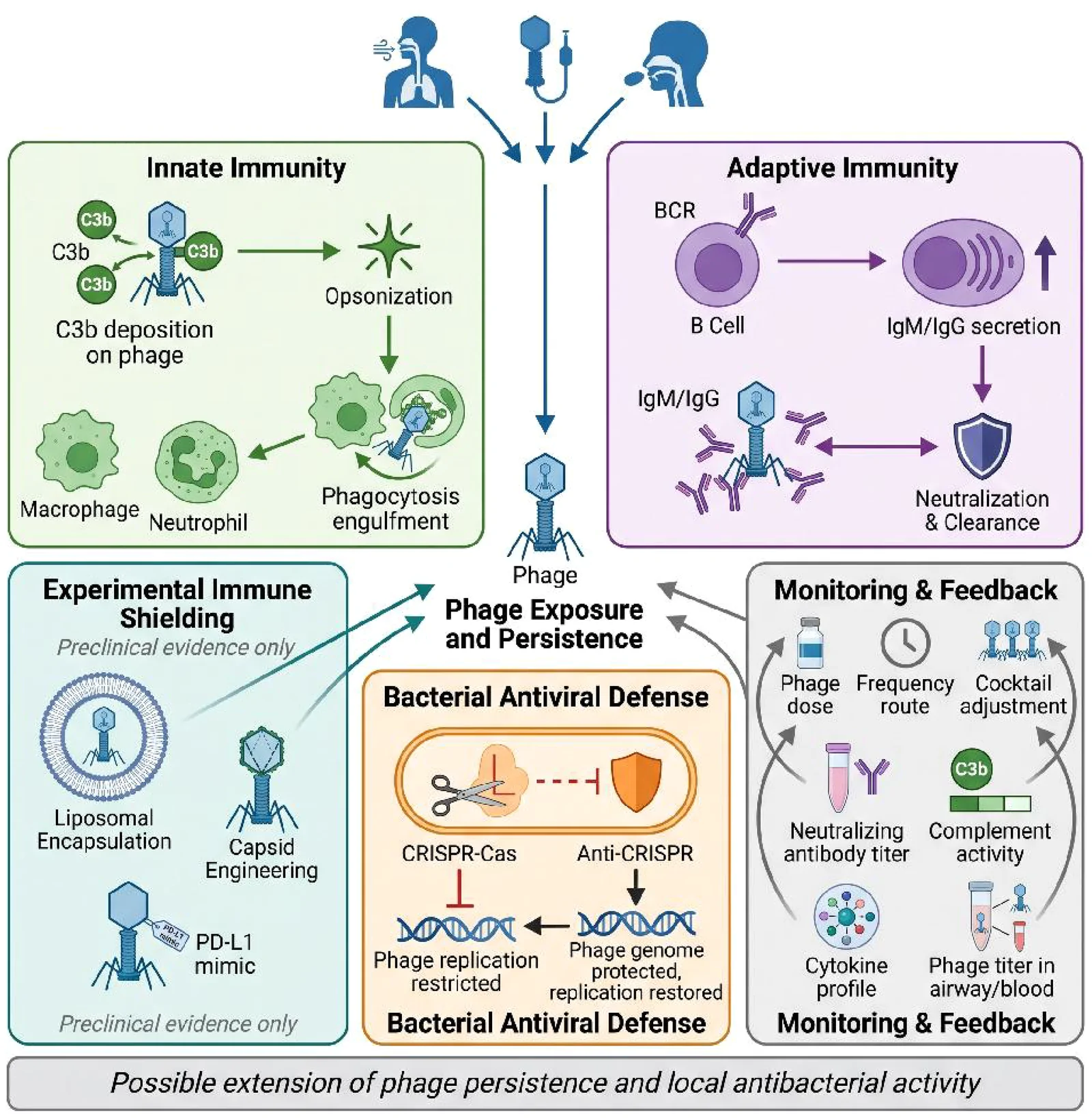
Host immune barriers, bacterial antiviral defense, and monitoring strategies in pulmonary phage therapy. Phages enter via inhalation, intravenous, or oral routes and converge on a central “phage exposure and persistence” node. Five surrounding modules summarize the regulatory mechanisms in this chapter. (Upper-left, green) Innate immunity: complement C3b deposition opsonizes phages for clearance by macrophages and neutrophils. (Upper-right, purple) Adaptive immunity: BCR-driven B cells secrete anti-phage IgM/IgG, neutralizing phages and accelerating their clearance. (Lower-left, blue) Experimental immune shielding covers liposomal encapsulation, capsid engineering, and PD-L1 mimetic peptides (preclinical evidence only). (Lower-middle, orange) Bacterial antiviral defense: CRISPR-Cas restricts phage replication, while anti-CRISPR proteins protect the phage genome and restore replication. (Lower-right, gray) Monitoring and feedback: neutralizing antibody titer, complement activity, cytokine profile, and airway/blood phage titers inform adjustment of dose, frequency, route, and cocktail composition. A bottom bar summarizes that these combined strategies may prolong phage persistence and local antibacterial activity.

### 4.2 Impact of adaptive immune responses on phage therapeutic efficacy

Adaptive immunity constrains phage efficacy mainly through B cell-mediated antibody responses. Linear or conformational epitopes on phage capsid proteins are recognized by B cell receptors, which trigger plasma cell secretion of specific IgM and IgG antibodies (Ghosh et al., 2025). These antibodies weaken phage function in two ways: they bind phage receptor-binding proteins directly and block adsorption to bacterial receptors by steric hindrance, and they promote Fc-mediated opsonization that accelerates phagocytic clearance by macrophages and neutrophils (Kim et al., 2025). Longitudinal clinical studies have linked rising IgM titers to the exponential decline in phage blood concentrations, suggesting that early antibody responses narrow the therapeutic window (Chan et al., 2025). T cell help governs antibody class switching. Phage antigens processed by dendritic cells are presented to CD4+ T cells via MHC-II, and activated follicular helper T cells (Tfh) secrete IL-21, driving B cell differentiation into IgG-secreting cells; this prolongs antibody half-life and enhances neutralization capacity (Gangwar et al., 2022). Memory B cells then make subsequent antibody responses faster and stronger upon repeat dosing, which explains the exponential decline in phage activity observed after multiple administrations (de Boer et al., 2026). Bacterial mutations that evade phage lysis often alter surface antigens (e.g., LPS O-antigen loss) and can indirectly affect adaptive immune recognition (Yang et al., 2025). This three-way interplay is complex: the fitness costs bacteria incur (e.g., reduced virulence) may be exploitable by the host immune system, yet phage-antibody complexes can also trigger complement-mediated inflammatory responses that worsen pulmonary tissue damage (Rodriguez-Gonzalez et al., 2024). Optimizing dosing regimens (e.g., pulsed dosing to avoid peak antibody periods, or liposomal encapsulation to shield phages from immune recognition) is therefore a core strategy for overcoming adaptive immune barriers (Terzi et al., 2026).

### 4.3 Engineered phages and immune regulation

Gene editing technologies (e.g., CRISPR-Cas systems or homologous recombination) allow precise modification of the phage genome to regulate host immune responses (Peng et al., 2025). Two mechanisms are central. First, insertion of genes encoding immunomodulatory factors (e.g., IL-10, TGF-β, or chemokines) lets phages release these molecules *in situ* upon bacterial lysis, reshaping the local immune microenvironment, dampening excessive inflammatory responses, and promoting tissue repair (Zhang et al., 2026a). Second, modification of capsid proteins (e.g., deletion or replacement of immunodominant epitopes) reduces recognition by pattern recognition receptors (e.g., TLR9, NOD2), which diminishes innate immune cell phagocytosis and complement activation and prolongs phage residence in the lungs (Peng et al., 2025). To evade bacterial immunity, engineered phages can express anti-CRISPR proteins (e.g., AcrIIA4, AcrVA1) that bind the nucleic acid-binding domains of bacterial CRISPR-Cas effector complexes (e.g., Cas9 or Cas12a), blocking cleavage of foreign DNA and protecting the phage genome (Qin et al., 2022). This also enhances lytic efficiency against drug-resistant bacteria and, by limiting the release of intracellular components (e.g., LPS, peptidoglycan fragments) on lysis, reduces excessive stimulation of the host immune system and the risk of cytokine storms (Qin et al., 2022). Additional strategies intervene systemically in the phage-host immune network. Engineered phages expressing Fc-binding proteins (e.g., protein A or protein G), for instance, competitively bind circulating anti-phage antibodies, block Fc-mediated opsonophagocytosis, and delay adaptive immune clearance (Zhang et al., 2026a). Phage display can integrate immunosuppressive peptides (e.g., PD-L1 mimetic peptides) into the capsid surface to inhibit T cell activation, reduce antibody class switching and memory B cell formation, and create an immune tolerance window for long-term repeat dosing (Bhale et al., 2026). These approaches support personalized immune monitoring, in which real-time measurement of anti-phage antibody titers, complement activity, and cytokine profiles in patient serum guides adjustment of engineered phage dosing, frequency, and immunomodulatory module expression for precision immunotherapy (Chan et al., 2025).

## 5 Phage therapy and the respiratory microbiome

### 5.1 Phage selectivity and the commensal microbiota

A defining feature of phages that distinguishes them from broad-spectrum antibiotics is their narrow host range, which is governed by the specificity of receptor-binding proteins for strain- or species-specific surface receptors. Whereas broad-spectrum antibiotics can deplete large fractions of the airway commensal community and thereby promote dysbiosis and opportunistic overgrowth, well-matched phages are predicted to remove the targeted pathogen while leaving most bystander commensals intact (Chan and Chang, 2022). This selectivity is, however, context dependent rather than absolute: it depends on receptor expression, bacterial density, and local physicochemical conditions, and closely related commensal strains that share receptors can in principle be co-targeted. Direct measurements of how the airway microbiome responds to phage therapy in humans remain scarce, and most evidence for selectivity currently comes from *in vitro* and animal models. The closest direct evidence to date comes from a prospective case series of nine adults with CF, in whom sputum microbiome diversity remained unchanged during 7–10 days of nebulized phage therapy, consistent with selective pathogen depletion without bystander commensal loss (Chan et al., 2025); the sample was nonetheless small and the observation period short, and larger, longer longitudinal studies are still required. Longitudinal airway microbiome profiling before, during, and after phage treatment is therefore needed to confirm whether selectivity translates into durable protection of the commensal community in patients.

### 5.2 Remodeling the airway ecosystem: direct and indirect routes

Beyond direct killing, phage therapy can reshape the airway ecosystem through several indirect routes. Clearance of a dominant pathogen can open an ecological niche that is then reoccupied by commensals or by other opportunistic pathogens; the direction of this succession determines whether the intervention is durably beneficial. Phage resistance, when it arises, can carry fitness costs, such as loss of capsular polysaccharide or LPS modifications, that alter competitive dynamics within the community and may reduce pathogen virulence (Wang et al., 2025). Phages can also carry polysaccharide depolymerases that degrade the biofilm matrix and disperse bacteria, changing the spatial organization of the community and potentially exposing pathogens to antibiotics and host effectors (Chakraborty et al., 2024). These effects are not always beneficial: disruption of a stable community and release of intracellular components on lysis can, in principle, trigger inflammation, and the net impact on the airway ecosystem must be assessed case by case.

### 5.3 The host-microbiome immune axis and microbiome-modulating strategies

Phages interact with the host-microbiome immune axis at multiple levels. By modulating pathogen burden and, potentially, the abundance of immunomodulatory commensals, phage therapy may indirectly influence local inflammation and mucosal immunity, in addition to the direct innate and adaptive responses to the phages themselves described in Section 4. Engineering extends the microbiome-modulating toolkit: phages expressing immunomodulatory factors, anti-CRISPR proteins, or depolymerases can be designed not only to kill pathogens but also to steer community composition and immune tone (Peng et al., 2025; Qin et al., 2022). Framing phage therapy as a microbiome-modulating intervention therefore shifts the therapeutic goal from simple pathogen eradication toward the restoration of a health-associated airway community. This framing also suggests new readouts for clinical trials, including longitudinal microbiome composition, community diversity, and functional metagenomic markers, alongside conventional microbiological and clinical endpoints.

## 6 Clinical case accumulation and translational pathway exploration

Clinical evidence for phage therapy in pulmonary infections remains limited and heterogeneous (**Table 1**). A 2025 systematic review identified only 18 human studies involving 70 patients with respiratory or pulmonary infections (Sarkodie-Addo et al., 2025) and highlighted substantial heterogeneity in study design, treatment protocols, phage preparations, administration routes, and outcome measures. The clinical literature remains dominated by case reports and case series, with only a small number of controlled clinical trials. Throughout this section, we therefore distinguish preclinical evidence, case-based clinical evidence, observational studies, and controlled clinical trials, and we qualify statements on efficacy accordingly. Reported outcomes should be interpreted cautiously, because most available data come from single cases or small series without comparator arms, and publication bias toward successful cases is likely.

**Table 1:** Human case reports and case series of phage therapy for drug-resistant bacterial pulmonary infections.

Patient(s)	Underlying pulmonary disease	Pathogen (resistance phenotype)	Phage composition	Route	Dose	Duration	Concomitant antibiotics	Microbiological outcome	Clinical outcome	Resistance emergence	Immune neutralization	Adverse events	Evidence type
1 (15-y girl with CF, status post bilateral lung transplant)	Disseminated *M. abscessus* infection with pulmonary involvement (post-transplant immunosuppression)	*M. abscessus* (resistant to all antibiotics tested)	Muddy + BPsΔ33HTH_HRM10 + ZoeJΔ45 (one natural and two engineered phages)	Intravenous every 12 h + topical application to sternal wound and skin lesions	~1 × 10^9^ PFU per phage per dose	32 weeks	Not reported (prior multidrug regimens had failed)	No phage-resistant isolates detected in serial specimens	Sternal wound closure, improved liver function, substantial resolution of skin nodules	None observed	Weak anti-phage antibody response without neutralizing activity	None significant; therapy well tolerated	Case report, compassionate use (Dedrick et al., 2019)
2 (9 adults with CF, median age 32 y)	Cystic fibrosis with chronic *P. aeruginosa* infection refractory to standard antibiotics	*P. aeruginosa* (7 MDR, 2 pan-resistant)	Personalized cocktail of 2–3 phages (n = 6) or single phage (n = 3), including OMKO1, LPS-5, TIVP-H6	Nebulized (jet nebulizer)	1 × 10¹^0^ PFU total per day, twice daily (inpatients) or once daily (outpatients)	7–10 days	6 patients on intravenous antibiotics at initiation; 3 had recently completed a course	Significant decline in sputum *P. aeruginosa* density (P < 0.05); sputum microbiome diversity unchanged	ppFEV1 improved (median +6% at days 21–35, P = 0.004)	Yes — phage-resistant clones in all patients, associated with fitness trade-offs (restored antibiotic susceptibility or reduced virulence)	Not assessed	Well tolerated; transient low-grade fever and malaise in 4/5 outpatients on days 2–3	Prospective single-arm case series, compassionate use, n = 9 (Chan et al., 2025)
3 (83-y man)	No chronic lung disease; interstitial pneumonia with ARDS on mechanical ventilation	*P. aeruginosa* (carbapenem-resistant, ST260; susceptible only to colistin and tobramycin)	Cocktail 1: PaSz-1_45_92k + PaZh_1 (days 1–14); cocktail 2: PaSz-1_45_92k + PaZh_1 + PAL9 (days 15–56)	Nebulized (jet nebulizer)	6 × 10^9^ PFU/dose twice daily (cocktail 1); 9 × 10^9^ PFU/dose (cocktail 2)	8 weeks (56 days)	Colistin (early course, discontinued for nephrotoxicity)	Progressive decline in sputum bacterial load without eradication (late isolates considered colonization); viable sputum phage titer up to 10^7^ PFU/mL	Inflammatory markers normalized; oxygenation improved; pleural effusion reduced on day-90 CT; renal function recovered	Yes — sequential isolates with partial phage resistance; final isolate fully resistant but with attenuated virulence, motility, and biofilm formation	Not assessed	None attributed to phage therapy (renal impairment attributed to colistin)	Case report (Yang et al., 2025)
4 (87-y woman)	COPD with severe pneumonia, ARDS, and renal failure on mechanical ventilation	*A. baumannii* (XDR; susceptible only to polymyxin B)	Single novel lytic *Caudoviricetes* phage (isolated from hospital sewage)	Nebulized	5 × 10^9^ PFU/mL twice daily	9 days (reported assessment period)	Fosfomycin 8 g q8h + amikacin 0.2 g q12h + polymyxin B 500,000 U q12h, all intravenous	Sputum cultures negative from day 4; mNGS pathogen burden reduced ~52-fold; ARGs reduced from 29 to 4	Hypercapnia resolved by day 4; CRP, ESR, and renal function improved	Not monitored *in vivo* (rapid tolerance to phage alone observed *in vitro*)	Not assessed	None; therapy well tolerated	Case report, compassionate use (Zhang et al., 2026b)

Preclinical (animal and *in vitro*) evidence is described separately in the text and is not presented as clinical cases. Fields not reported in the primary sources are labeled “Not reported” or “Not assessed” rather than inferred.

ARDS, acute respiratory distress syndrome; ARG, antimicrobial resistance gene; CF, cystic fibrosis; COPD, chronic obstructive pulmonary disease; CRP, C-reactive protein; CT, computed tomography; ESR, erythrocyte sedimentation rate; FEV1, forced expiratory volume in 1 second; IV, intravenous; MDR, multidrug-resistant; mNGS, metagenomic next-generation sequencing; PFU, plaque-forming unit; ppFEV1, percent predicted FEV1; q8h/q12h, every 8/12 hours; XDR, extensively drug-resistant.

Primary sources: Dedrick et al., Nat Med 2019;25:730–733 (doi: 10.1038/s41591-019-0437-z). Chan et al., Nat Med 2025;31:1494–1501 (doi: 10.1038/s41591-025-03678-8). Yang et al., Sci Rep 2025;15:33512 (doi: 10.1038/s41598-025-17510-3). Zhang et al., Front Cell Infect Microbiol 2026 (doi: 10.3389/fcimb.2026.1851410).

### 6.1 Clinical success cases of personalized phage therapy

Phage therapy has shown promise in the personalized treatment of MDR bacterial pulmonary infections, although this evidence is predominantly case-based. In patients with cystic fibrosis (CF) and chronic pulmonary infection with MDR *P. aeruginosa*, conventional antibiotics are often ineffective, whereas nebulized inhalation of precisely matched phage cocktails has been associated with clinical benefit in the reported cases. A scoping review of 23 human case studies found that 87.5% of patients showed improvement or clearance of infection after phage therapy (Sukri et al., 2026). Pulmonary function parameters (e.g., FEV1) improved, sputum bacterial burden declined, and no serious adverse events were reported, consistent with the high specificity of phages and their replication in the pulmonary microenvironment (Iszatt et al., 2021). Mycobacterial infections (e.g., *M. abscessus*, *Mycobacterium avium*-intracellulare) have also in individual cases been treated. In a landmark case, a 15-year-old patient with cystic fibrosis and a disseminated, extensively drug-resistant *M. abscessus* infection following bilateral lung transplantation was treated with a three-phage cocktail comprising the natural phage Muddy and two engineered phages, BPsΔ33HTH_HRM10 and ZoeJΔ45, administered intravenously and topically; this was associated with sternal wound closure, improved liver function, and substantial resolution of infected skin nodules (Dedrick et al., 2019). The case provided key evidence that even pathogens highly resistant to conventional antibiotics can be controlled in individual cases through precise phage selection and engineering. These cases suggest that personalized phage therapy may have a role in complex, refractory MDR pulmonary infections, and that success depends on precise pathogen identification, efficient phage selection, and rational dosing (Kim et al., 2025).

### 6.2 Limitations and improvement strategies of phage therapy in complex infections

Phage therapy has succeeded in many MDR bacterial infections, but not every clinical attempt has worked, and the failures deserve equal attention. One patient with disseminated *Stenotrophomonas maltophilia* infection received 12 days of combined intravenous and intraperitoneal phage therapy; blood cultures converted to negative, yet intraperitoneal fluid cultures remained positive and the patient died of multi-organ failure (Cullen et al., 2024). The case shows that in complex, multifocal, biofilm-associated infections, phage therapy may not fully clear the focus of infection, especially in intensive care settings where the therapeutic window is narrow (Poniatovskyi et al., 2025). Biofilm formation is a central cause of treatment failure: bacteria within biofilms use exopolysaccharide matrices to create physical barriers that reduce phage permeability and killing efficiency (Moghadam et al., 2026). Rapid emergence of phage resistance is another major cause. Under phage selective pressure, bacteria can evade infection through mutations that alter surface receptors (e.g., capsular polysaccharide, LPS), allowing resistant strains to regrow (de Villiers de la Noue et al., 2025). In preclinical models of *K. pneumoniae* phage therapy, for instance, bacterial regrowth was observed after initial treatment efficacy, which illustrates how common phage resistance is (Shein et al., 2024). Treatment protocols must therefore be optimized to improve success rates: extending treatment duration to suppress bacterial growth, adding biofilm-disrupting agents (e.g., DNase, EDTA) to improve phage penetration, and monitoring host immune responses to prevent efficacy loss through immune clearance (Chung et al., 2023). Phage-antibiotic combinations can also suppress bacterial regrowth and reduce the probability of resistance, although the outcome is context dependent: it varies with antibiotic class, concentration, sequence and timing of administration, bacterial growth state, and phage replication kinetics, and antagonism is possible in some combinations (Tarasenko et al., 2025; Zhang et al., 2026b). These failures indicate that phage therapy is not a panacea; success depends on understanding the infection microenvironment, bacterial biology, and host immune status. Standardized protocols, real-time monitoring tools, and combination strategies will need further development. .

## 7 Conclusion

Phage therapy for MDR bacterial pulmonary infections has moved from laboratory proof-of-concept to early clinical evaluation, while remaining an investigational strategy dominated by case-based evidence. In this review, we have reframed phage therapy as a precision microbiome-modulating strategy rather than merely another antimicrobial. From this perspective, the central task is to selectively deplete MDR pathogens and steer the airway ecosystem back toward a health-associated community, using precise pathogen matching, engineered pulmonary delivery, and management of the host immune response as integrated components of a single strategy. Precise pathogen identification and phage matching remain the cornerstones of efficacy, but matching efficiency and success rates vary across studies, pointing to the need for standardized phage banks and rapid screening workflows. Engineering advances in inhalation formulations have overcome the physical barriers to localized pulmonary delivery, while recognition and clearance of phages by the host immune system constitute the main barrier to durable efficacy. Studies disagree on the intensity and duration of immune responses, so dosing must move from a one-size-fits-all approach toward individualized regimens guided by immune monitoring data. The clinical evidence base remains limited and heterogeneous: it is dominated by case reports and case series, with only a small number of controlled trials, so claims of efficacy must remain appropriately qualified. Major evidence gaps include the absence of longitudinal airway microbiome profiling in treated patients, the lack of standardized protocols for combination dosing, and the need for randomized controlled trials with prespecified microbiome and clinical endpoints. Looking ahead, engineered phages and their derivatives, combined with model-driven dose optimization and novel nanodelivery technologies, could carry phage therapy from experimental salvage treatment toward mainstream clinical practice. That step requires interdisciplinary collaboration, rigorous randomized controlled trials, and integration of real-world evidence, ultimately shifting phage therapy from case-by-case miracles to standard therapy grounded in microbiome-informed precision.
